# Isolation and Characterization of Multipotent CD24+ Cells From the Renal Papilla of Swine

**DOI:** 10.3389/fmed.2018.00250

**Published:** 2018-09-19

**Authors:** David M. Burmeister, Matthew K. McIntyre, Robbie K. Montgomery, Belinda I. Gómez, Michael A. Dubick

**Affiliations:** Damage Control Resuscitation, United States Army Institute of Surgical Research, San Antonio, TX, United States

**Keywords:** stem cells, papillae, pig models, transplantation, progenitor cells, swine, kidney

## Abstract

Over 100,000 patients in the United States are currently waiting for a kidney transplant. With just over 10,000 cadaveric kidneys transplanted annually, it is of the utmost importance to optimize kidney viability upon transplantation. One exciting avenue may be xenotransplantation, which has rejuvenated interest after advanced gene editing techniques have been successfully used in swine. Simultaneously, acute kidney injury (AKI) is associated with high morbidity and mortality and currently lacks effective treatment. Animal models have been used extensively to address both of these issues, with recent emphasis on renal progenitor cells (RPCs). Due to anatomical similarities to humans we aimed to examine progenitor cells from the renal papillae of swine kidneys. To do this, RPCs were dissected from the renal papillae of healthy swine. Cell surface marker expression, proliferation, and differentiation of the RPCs were tested *in vitro*. Additionally, a mixed lymphocyte reaction was performed to examine immunomodulatory properties. RPCs displayed spindle shaped morphology with limited self-renewing capacity. Isolated RPCs were positive for CD24 and CD133 at early passages, but lost expression with subsequent passaging. Similarly, RPCs displayed myogenic, osteogenic, and adipogenic differentiation capacities at passage 2, but largely lost this by passage 6. Lastly, direct contact of RPCs with human lymphocytes increased release of IL6 and IL8. Taken together, RPCs from the papilla of porcine kidneys display transient stem cell properties that are lost with passaging, and either represent multiple types of progenitor cells, or a multipotent progenitor population. In instances of ischemic insult, augmentation of/with RPCs may potentiate regenerative properties of the kidney. While the use of swine for transplantation and ischemia studies confers obvious advantages, the populations of different progenitor cell populations within pig kidneys warrants further investigation. Ultimately, while gene editing techniques enhance the potential for xenotransplantation of organs or cells, the ultimate success of this strategy may be determined by the (dis)similarities of RPCs from different species.

## Introduction

While roughly 17,000 kidney transplants are performed annually in the United States, over 100,000 Americans are currently waiting for a kidney transplant (1). With such a paucity of donor kidneys, there is a great need to maximize organ preservation in order to prevent rejection and improve long term outcomes post-transplantation (2). To realize these optimal conditions in the face of precious few human kidneys, both small (3) and large (4) animal models have provided valuable insight. Moreover, the potential for filling this need with xenotransplantation has been made even more possible with advanced gene editing techniques (5) and other burgeoning technologies that render clinical trials utilizing pig kidneys closer than ever (6).

Separately, acute kidney injury (AKI) disrupts the kidneys' blood filtering function and increases mortality, hospital length of stay, and hospital costs (7). Molecular mechanisms driving AKI have also been informed by a variety of animal models from chemical induction to trauma (8, 9). However, there remains a growing need to address the lack of effective treatments for AKI. More recent advances in stem cell biology have led to the examination of various cell populations in animal models of renal disease. For example, a variety of cell types have been used to examine therapeutic efficacy in both AKI and renal transplantation models (10–12).

Alternatively, it has been shown that renal recovery following ischemia-reperfusion injury is influenced by a population of intrinsic renal cells rather than extra-renal mesenchymal cells (13, 14). Indeed, there have been several studies that have attempted to localize the renal stem cell niche via such methods as BrdU label retention. These studies have resulted in different claims of renal stem cells from locations such as proximal tubules, renal papillae, parietal epithelial cells, glomeruli, or Bowman's capsule (15–20). Recent consensus seems to have been reached that regeneration of the tubular epithelium occurs from cells that originate in the tubules. However, debate still ensues as to whether or not these cells are resident stem cells in the proximal tubules, or if all terminally differentiated tubular epithelial cells have the ability to dedifferentiate and proliferate to repopulate the tubular epithelium (21, 22). It is likely that different progenitor cell populations are involved with different reparative processes depending on the etiology of renal dysfunction.

Along these lines, the controversy over the existence and localization of these cells has recently been addressed by the theory that multiple populations of progenitor cells exist within the adult kidney (23). This notion was further evidenced in a recent report by Oliver et al. which used genetic lineage tracing of Zfyve27 to show that different locations in the kidney are populated by different progenitor cell sets (24). One such location for renal progenitor cells (RPCs) is the renal papilla (19), which has been shown to yield such cells in a recent study in rodents (25). While many studies have been carried out in small animals, further pre-clinical studies in large animals are needed (26). In particular, the kidneys of domestic swine have several characteristics that give certain advantages for comparison to human renal (patho) physiology. Swine kidneys are pyramidal/multi-lobular with similar vasculature to human kidneys (4). Kidney and body size, as well as renal biochemical markers creatinine and blood urea nitrogen (BUN), are also comparable between pigs and humans. To examine this progenitor cell population in pigs, we isolated renal papilla from porcine kidneys and examined their: proliferative ability; capacity to differentiate into multiple lineages; cell surface expression of CD24 and CD133 and; immunomodulatory properties. We find that these cells are heterogeneous in nature, have limited self-renewing capacity and stem-cell like properties that are lost with subsequent passaging.

## Materials and methods

### Animals

Seven Yorkshire pigs (Midwest Research Swine, Gibbon, MN) weighing 68.0 ± 4.9 kg were used in this study. To ensure health of the animal and the kidney, blood samples were taken prior to euthanasia for analysis of blood chemistry and complete blood count (CBC). All procedures were approved by the Regulatory Research Compliance Division of the US Army Institute of Surgical Research (USAISR). Specifically, a tissue sharing request to the Animal Care and Use Committee from USAISR was completed. This research was conducted in compliance with the Animal Welfare Act, the implementing Animal Welfare Regulations, and the principles of the Guide for the Care and Use of Laboratory Animals, National Research Council. The facility's Institutional Animal Care and Use Committee approved all research conducted in this study. The facility where this research was conducted is fully accredited by AAALAC International. For protein, creatinine, and BUN measurement, blood was collected into a BD Vacutainer tube with Lithium Heparin (Becton Dickinson and Company, Franklin Lakes, NJ) and centrifuged at 1,200 g for 10 min. Samples were analyzed on a Siemens Dimension® Xpand™ Plus clinical Chemistry system. For CBC analysis, specimens were collected in a BD Vacutainer tube containing K2 EDTA (Becton Dickinson and Company, Franklin Lakes, NJ) and analyzed with the Abbott Cell-Dyn® 3700 system.

### RPC isolation

**Figure 1:**
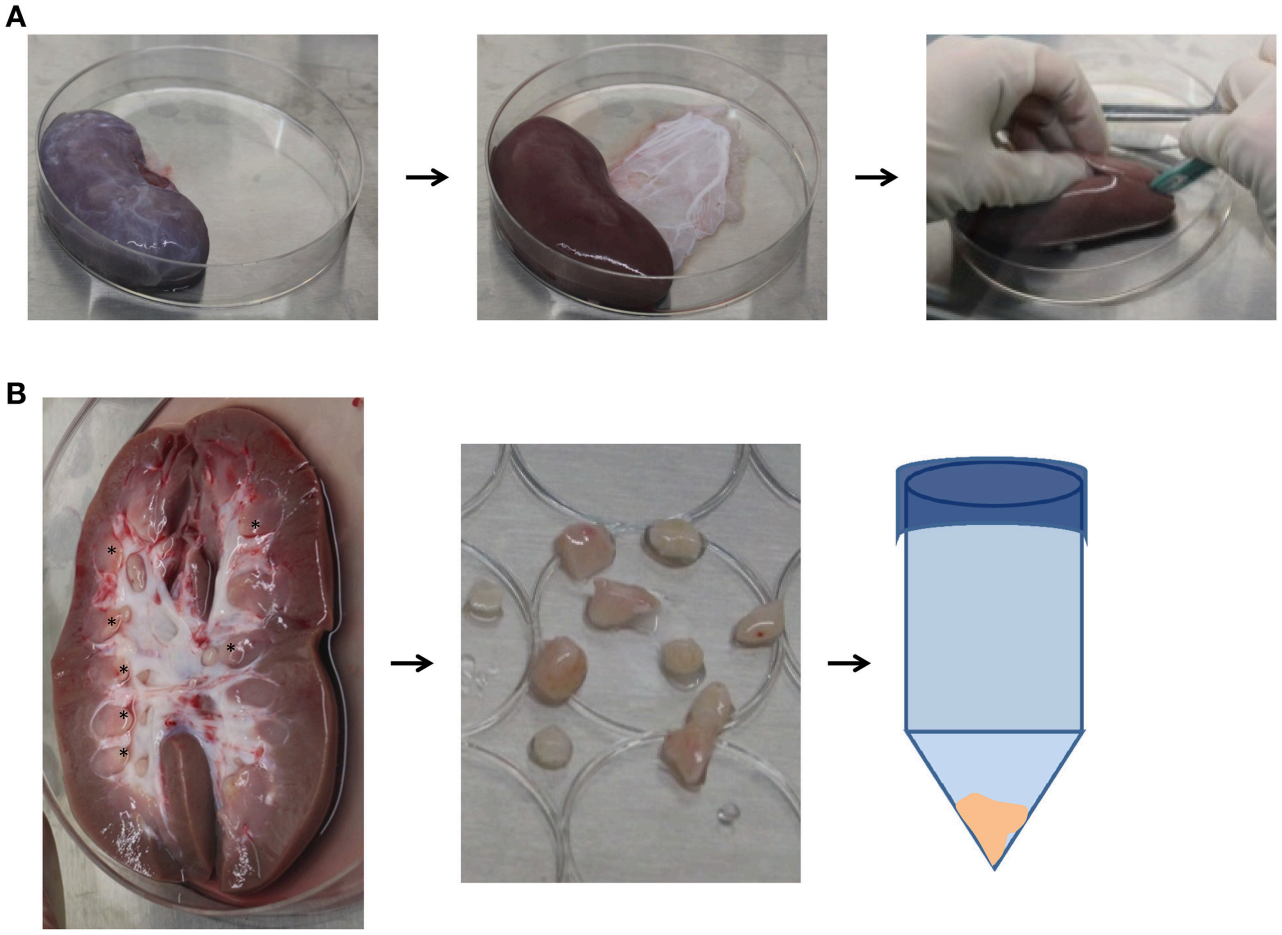
Isolation procedure for papillary renal progenitor cells. **(A)** Sterile blunt dissection of excised porcine kidneys was done via removal of the renal capsule, and a subsequent coronal incision. **(B)** Visible papillary tips (asterisks) were cut away from the remainder of the kidney and minced with scissors, where they were then incubated in 2mg/mL collagenase at 37°C for 30 min. After enzymatic digestion, papillae were centrifuged, the supernatant was discarded, and cell pellet plated.

Animals were euthanized with pentobarbital sodium. Using sterile technique, kidneys were excised, and placed in Hanks Balanced Salt Solution (HBSS) (Gibco, Gaithersburg, MD) immediately post-mortem for isolation of the renal papilla as previously described (27) and shown in Figure 1. Briefly, the renal capsule was removed and a longitudinal incision along the coronal axis was made to identify and remove the renal papillae. Papillae were washed with HBSS and minced, before being digested in Collagenase type II (Gibco, 2 mg/mL in HBSS) for 30 min at 37°C. Samples were centrifuged at 1,900 rpm for 10 min and the pellet was re-suspended in Mesenpro RS™ growth media (Gibco) containing supplement, l-glutamine (Gibco), and 1–2% antibiotics/antimycotics (AB/AM, Gibco). Cells were plated on T-75 flasks (Corning, Corning, NY) and incubated at 37°C with 5% CO2. Non-adherent cells were removed at day 2 and media was changed every 2-3 days. The remaining adherent cells were defined as RPC and allowed to reach 80% confluency after which they were passaged with 0.25% Trypsin/EDTA (Gibco) for 5 min before neutralization with Mesenpro RS™ growth media.

### Proliferation

At passages 2 and 6, 1 × 10^4^ RPCs were plated into triplicate wells of a 96-well plate and grown in Mesenpro RS™ media with supplements listed above. At days 1, 2, 3, and 7 post-plating, media was removed and replaced with 4% paraformaldehyde (PFA) until all cells were fixed, then plates were stored at −80°C until analysis. A standard curve was generated on each plate by seeding increasing amounts of cells in triplicate, allowing 4 h for adherence and then fixing cells with 4% paraformaldehyde (PFA) and subsequently freezing. CyQUANT Cell Proliferation Assay Kit (Molecular Probes, Eugene, OR) was performed according to manufacturer's protocol for determining the density of cells.

### Immunofluorescence microscopy

At passages 2 and 6, 5 × 10^3^ RPCs were seeded on a 96-well plate in triplicate and allowed to adhere for 4 h in growth media. Cells were fixed with 4% PFA in HBSS for 15 min and subsequently washed with HBSS. Cells were then blocked with 1% BSA (Sigma Aldrich, St. Louis, MO) with 0.1% Triton X-100 (Thermo Fisher, Waltham, MA) for 1 h at RT. The following primary antibodies were used for immunocytochemistry: CD24 (Bioss, 1:100 dilution), CD133/Prom1 (Genway, GWB-MW170D, 1:50 dilution), NFATC-1 (LSBio, LS-C40633, 1:10 dilution), CD90 (BD Biosciences, 555595, 1:10 dilution), and CD45 (R & D Systems, FAB1430P, 1:10 dilution); all diluted in blocking buffer for 3 h at room temperature. Cells were then washed, blocked again with BSA solution for 1 h, and subsequently incubated with either Donkey anti-rabbit Alexa Fluor 488 secondary antibodies (Thermo, 1:200 dilution) for 1 h at RT. Cells were again washed, and incubated with HBSS containing 0.5 μg/mL DAPI.

### Flow cytometry

At passages 2 and 6, 5 × 10^5^ RPCs were collected as described above and re-suspended in 100 μL of HBSS. The same antibodies listed above for CD24 (1:100 dilution), CD133/Prom1 (1:20 dilution), and NFATC-1 (1:20 dilution) were conjugated with Zenon™ Alexa Fluor™ 647 or 488 anti-rabbit IgG labeling kit according to manufacturer protocol. An unstained population of RPCs, and one incubated with the Zenon reagents served to set the size and fluorescence gating. After mixing, 100 μl of the entire solution was then added to cells and incubated in the dark for 1 h. Cells were then washed once and flow cytometry was performed using a BD FACSCanto (BD biosciences, San Jose, CA) by collecting 10,000 events/sample. Analysis was performed with BDFACS Diva software.

### Adipogenic differentiation

Adipogenic differentiation was performed as previously described for other mesenchymal stem cells (MSCs) (28). All chemicals were purchased from Sigma-Aldrich unless otherwise specified. Briefly, passage 2 and 6 RPCs were seeded on T-25 flasks at a density of 1 × 10^4^ cells/cm^2^ containing high glucose DMEM containing 10% FBS, Ab/Am, L-glutamine, 0.5 mM Isobutyl-methyl xanthine, 200 μM indomethacin, 10 μM insulin, 1 μM dexamethasone, and 10 μM ciglitazone and incubated at 37°C supplemented with 5% CO2. Media was changed every 2–3 days and cells were fixed with 4% PFA after 2 weeks. After all passages were collected, cells were washed with dH20 and incubated with 60% isopropanol before incubation with Oil Red O solution.

### Osteogenic differentiation

Osteogenic differentiation was performed as previously described for other MSCs (28). Briefly, passage 2 and 6 RPCs were seeded on T-25 flasks at a density of 1 × 10^3^ cells/cm^2^ containing α-MEM containing 10% FBS, Ab/AM, L-glutamine, 50 μM Ascorbate-2-phosphate, 10 mM b-glycerophosphate, 10 ng/mL bone morphogenic protein 2 (R&D Systems), and 0.1 μM dexamethasone and incubated at 37°C supplemented with 5% CO2. Media was changed every 2-3 days and cells were fixed with 4% PFA after 2 weeks, after which cells were washed with HBSS [no Ca^++^)] and stained with Alzarin red solution (Millipore).

### Myogenic differentiation

Myogenic differentiation was performed as previously described for other MSCs (28). Briefly, passage 2 and 6 RPCs were seeded on T-25 flasks at a density of 2 × 10^3^cells/cm^2^ that were previously coated with 10 uL/cm^2^ Matrigel (Corning) diluted 1:6 in myogenic differentiation media containing low glucose DMEM supplemented with 5% Horse serum, AB/AM, 50 μM hydrocortisone, and 0.1 μM dexamethasone and incubated at 37°C supplemented with 5% CO2. Media was changed every 2–3 days and cells were fixed with 4% PFA after 2 weeks. After all passages were collected, cells were blocked with 1% BSA containing 0.1% Triton X-100 for 1 h, and incubated overnight at 4°C with anti-Myosin Heavy Chain antibody (R&D Systems) (diluted 1:50) in 2% goat serum. The following day cells were washed with PBS and blocked for 1 h in 5% goat serum and then incubated for 2 h at room temperature in Alexa-488 goat anti-mouse antibody (Life Technologies; 1:400) in 2% goat serum.

### RPC effect on porcine PBMCs

To examine the direct and paracrine effects of RPCs on peripheral blood mononuclear cell (PBMC) cytokine production, a mixed lymphocyte reaction was performed. PBMCs were isolated from healthy donor pigs (*n* = 4) in duplicate using Ficoll-Paque PLUS (GE Healthcare, Uppsala, Sweden) according to manufacturer protocol. Lymphocytes were washed with HBSS and re-suspended in RPMI-1640 medium (ATCC modification, Gibco) supplemented with 10% FBS (Gibco), and 1% AB/AM (Gibco). 2 × 10^5^ PBMC were seeded on 24 well plates. In duplicate wells 2 × 10^5^ passage 2 RPCs were then added either in the well directly, or into 0.4 μm pore size polycarbonate transwell inserts, or control wells were made with no RPCs. To stimulate the PBMCs 5 μg/mL of phytohaemagglutinin-L (PHA) was added and the cells were allowed to incubate for 3 days at 37°C supplemented with 5% CO2. Cell supernatant was collected and stored at −80°C until analysis. Supernatants were analyzed with a porcine-specific MILLIPLEX Cytokine/Chemokine magnetic bead panel kit (EMD Millipore, PCYTMG-23K-13PX), which was performed according to manufacturer protocol.

### Statistical analysis

Statistical evaluations were performed using GraphPad Prism software (GraphPad Software, San Diego, CA). For the proliferation assay, a two-way ANOVA was performed to examine the effect of time and passage, with Bonferroni *post-hoc* testing. For the PBMC cytokine release, non-normally distributed data dictated that a Kruskal Wallis test with Dunn's Multiple Comparisons was employed. Technical replicates were averaged to produce a single value for biological replicates, which are expressed as the arithmetic mean ± SEM, and *p* < 0.05 were considered significant.

## Results

### Morphology and growth of porcine RPCs

We isolated renal papillae from porcine kidneys immediately post-mortem under surgical sterile conditions (Figure 1). Table 1 shows circulating biochemical values from the animal just before euthanasia. All animals displayed creatinine, BUN, total protein, and creatinine kinase values within the normal range for swine, indicating that renal function was not compromised. Moreover, the values of circulating white blood cells were also within normal range, indicating overall health of the animal (Table 1).

**Table 1:** Various kidney function and leukocyte levels circulating in animals used for cell isolation.

**Measure**	**Unit**	**Mean ±SEM**
Creatinine	mg/dL	1.61 ± 0.09
Total protein	g/dL	6.68 ± 0.14
BUN	mg/dL	9.55 ± 0.60
Creatinine kinase	U/L	513.16 ± 78.21
Neutrophils	×10^3^/μl	3.19 ± 0.51
Lymphocytes	×10^3^/μl	6.72 ± 0.62
Monocytes	x10^3^/μl	0.31 ± 0.03
Eosinophils	x10^3^/μl	0.41 ± 0.06
Basophils	x10^3^/μl	0.05 ± 0.01

**Figure 2:**
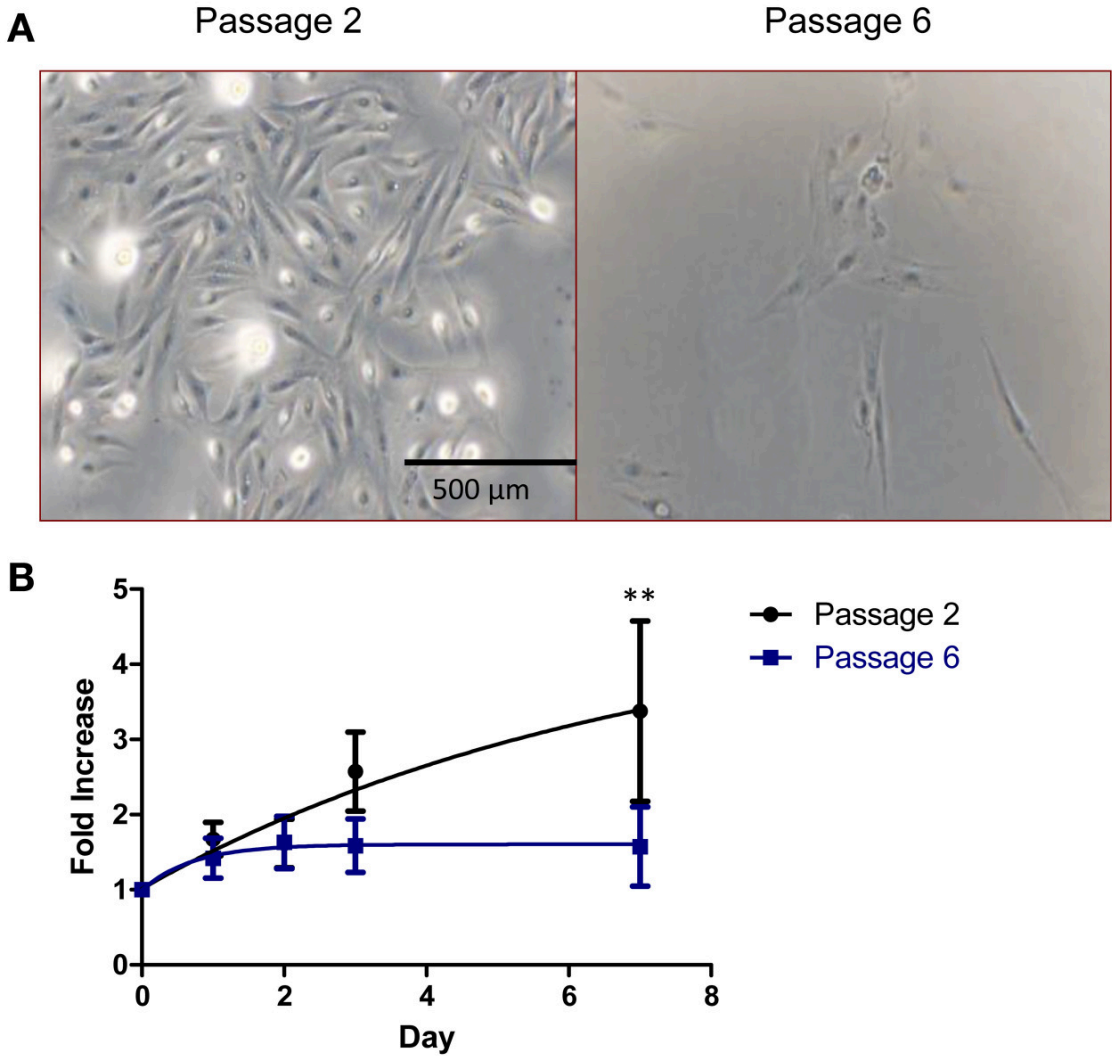
Cell morphology and growth kinetics of RPCs. **(A)** Brightfield images reveal that RPCs at low passages (passage 2) have a spindle-like morphology similar to mesenchymal stem cells, which is lost with subsequent passages. **(B)** Similarly, cell proliferation assays were performed, and the proliferative ability of RPCs at passage 2 is lost by passage 6, with statistical significance at day 7. Data expressed as mean ± SEM of 3 replicates. ^**^P < 0.01 from 6 to 7 kidneys, at passage 2 and passage 6, respectively.

As shown in Figure 2A, isolated RPCs exhibited classic mesenchymal spindle shaped morphology and propensity to adhere to standard tissue culture plastic. At early passages (passage 2 shown in Figure 2A) RPC routinely expanded until harvesting/passaging at 80% confluence. However with subsequent passaging, it became apparent that the self-renewing capacity of porcine RPCs is limited by passage 6. Quantification of proliferative capacity is demonstrated in Figure 2B for cells at passage 2 and passage 6, wherein RPCs at the later passage failed to divide. Two-way ANOVA revealed a significant effect of time (*P* = 0.0095) and passage (*P* = 0.0469) on RPC proliferation. The fold increase in cells was significantly higher for passage 2 RPCs at day 7 (*p* < 0.01, *n* = 6 for passage 2, and *n* = 7 for passage 6), but not quite significant for day 3 (*P* = 0.093). There was some heterogeneity in the longevity of primary cultures despite consistent isolation protocols. Specifically, we observed one population out of seven that continued to display a proliferative ability at passage 6, which is reflected in the variability.

### Stem cell markers present on RPCs

**Figure 3:**
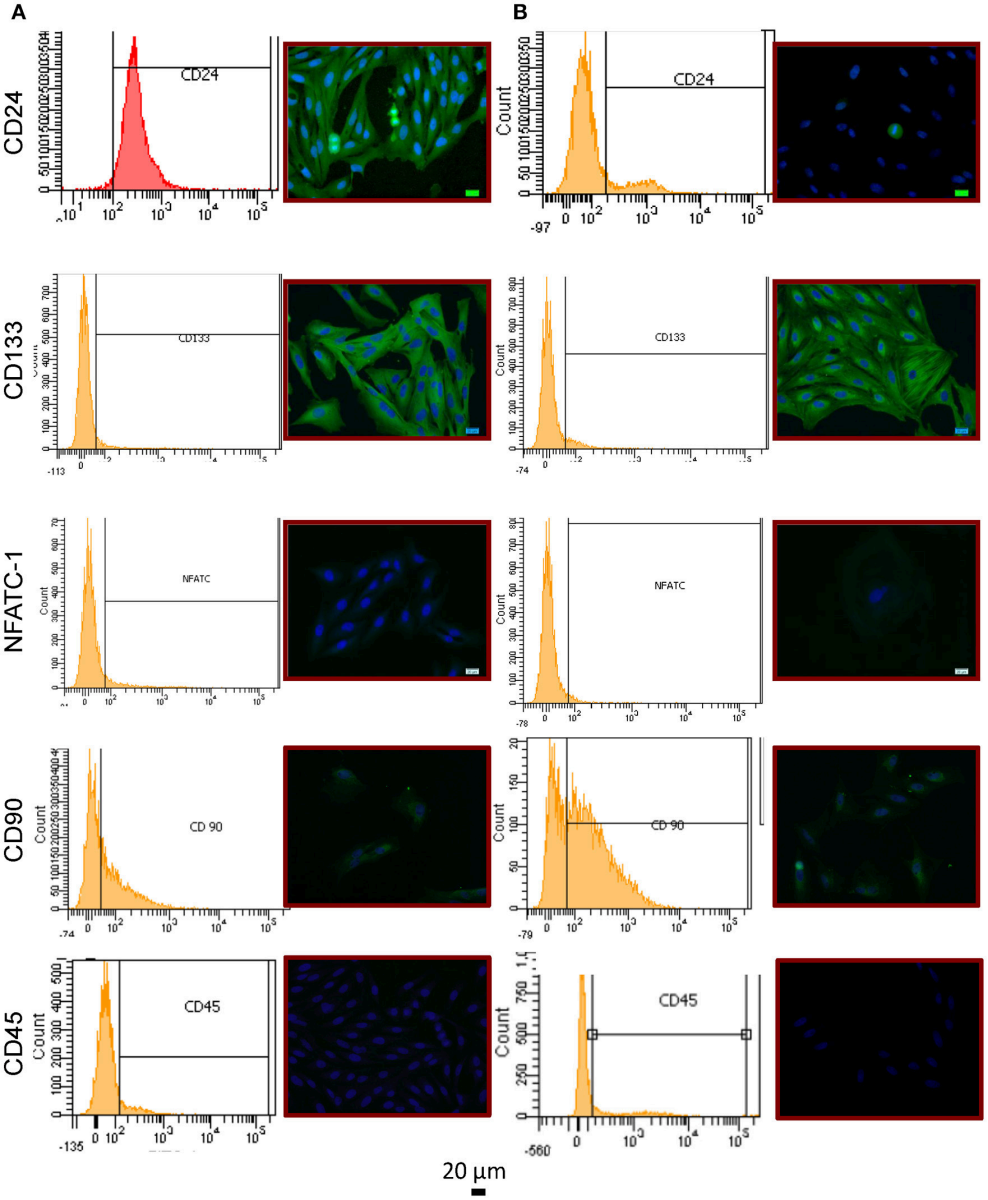
Immunophenotype of RPCs **(A)** Passage 2 cells showed expression of CD24 (top) via immunocytochemistry as well as flow cytometry. While CD133 expression (middle) was strongly evident with immunocytochemistry, the same antibody was not able to detect expression via flow cytometry. CD90 was shown to be weakly expressed, and not in all cells. NFATc1 (middle row) and CD45 (bottom row) were shown not to be expressed with either method. **(B)** By passage 6, CD24 expression was drastically reduced.

To examine whether RPCs expressed common renal “stem cell” population markers we performed immunocytochemistry and flow cytometry on CD24, CD133, and nuclear factor of activated T-cells 1 (NFATc1) (Figure 3). At passage 2, 96.3 ± 1.1% of isolated RPCs expressed CD24 staining which was confirmed with immunocytochemistry. However by passage 6, this percentage was drastically reduced to 23.8 ± 11.2%, (*P* = 0.0095) with a variability reflecting heterogeneity in that only one isolate still expressed significant CD24. Immunocytochemical staining for CD133 proved to be strong in RPCs at both passages 2 and 6, although by passage 6 there were isolated areas of striated staining apparent. However, flow cytometry analysis with the same antibody did not corroborate this, likely due to the lack of antibody suitability for flow cytometry. A minor amount of NFATc1 (6.85 ± 0.93%, and 4.75 ± 2.21% at passage 2 and 6, respectively) was expressed as determined by flow cytometry. Expression of NFATc1 was also largely not seen with immunocytochemistry. Similarly, CD45 expression was low 14.55 ± 1.97%, but higher than NFATc1. A certain degree of heterogeneity in stem cell marker expression was seen, as CD90 expression was 44.4 ± 2.6 % and 47.2 ± 6.0% at passages 2 and 6, respectively.

### Multipotent differentiation of RPCs

**Figure 4:**
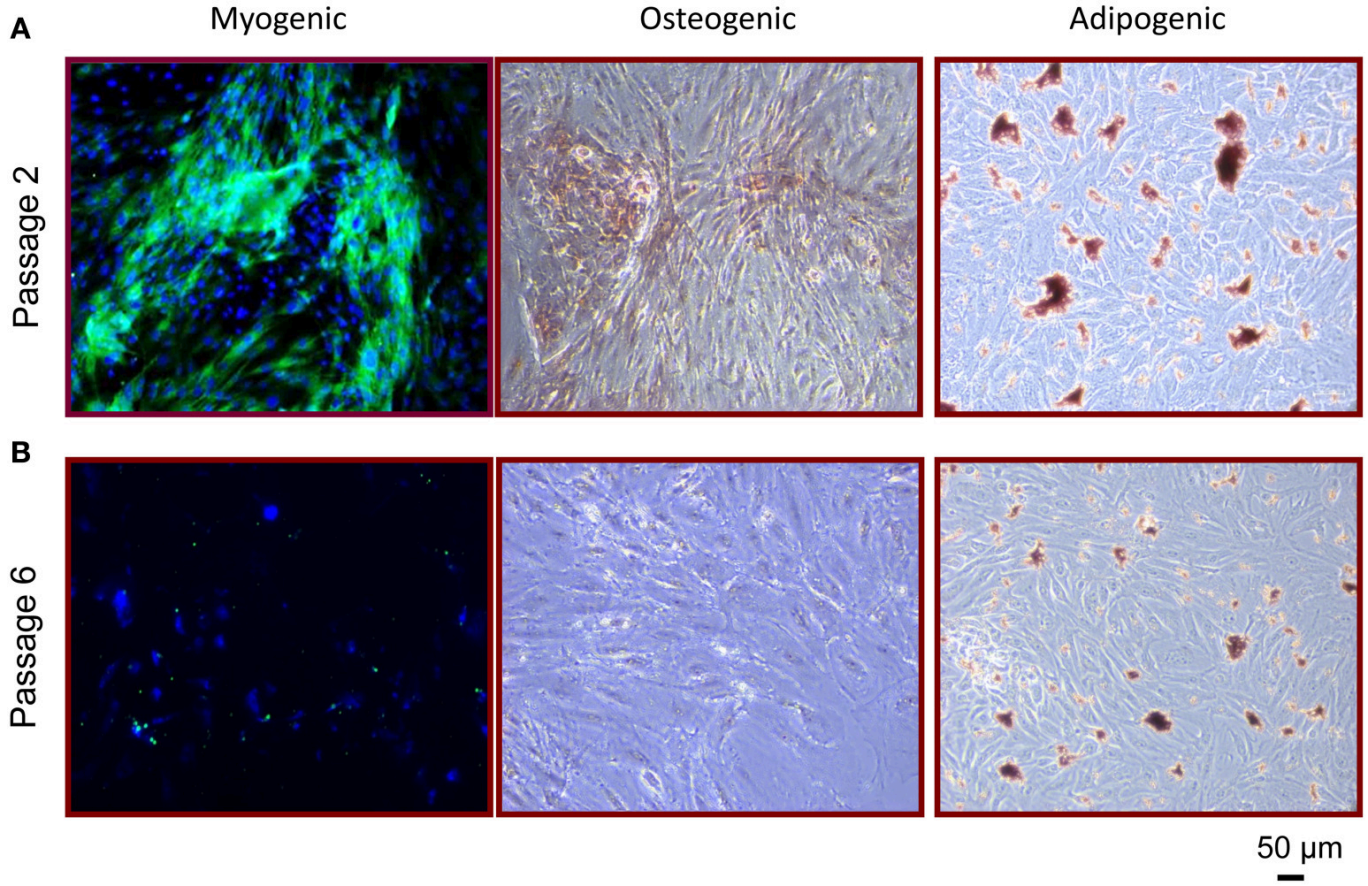
Multipotent differentiation potential of RPCs. **(A)** At passage 2, RPCs were able to differentiate into myogenic (left), osteogenic (middle), and adipogenic (right) lineages, which was not as apparent at passage 6 **(B)**.

To examine whether RPCs have the ability to differentiate into other cells of mesenchymal origin, we utilized previously established differentiation protocols as detailed in the Methods, to induce adipogenesis, osteogenesis, and myogenesis. As shown in Figure 4, isolated RPCs demonstrated multipotent ability in a passage-dependent manner. It is important to note that, at no point, did differentiation occur in all RPCs for any of these lineages, illustrating the heterogenous phenotype of these cells. RPCs introduced to myogenic differentiation media began to express levels of myosin heavy chain after 2 weeks at passage 2, while RPCs at passage 6 did not have this ability. Similarly, the Alizarin Red staining seen with culturing passage 2 RPCs in osteogenic media was not apparent at passage 6. While passage 2 RPCs did show a limited ability for adipogenesis in that respective media, the drop off in this differentiation capacity from passage 2 to passage 6 was not as marked as with the other two lineages.

### RPC's effect on mixed lymphocyte reactions

**Figure 5:**
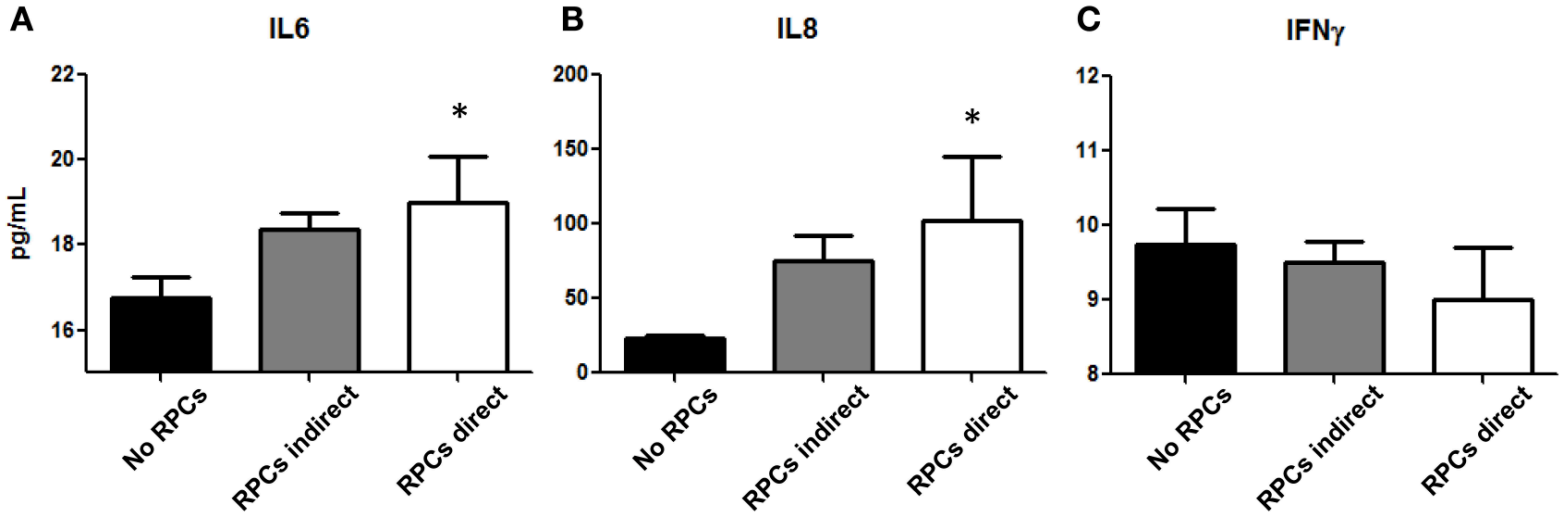
RPCs effect on Peripheral Blood Mononuclear Cells. Cytokines IL-6 **(A)**, IL-8 **(B)**, and IFNγ **(C)** released from isolated lymphocytes from healthy swine are altered with the addition of RPCs. Both IL-6 and IL-8 (CXCL-8) are slightly increased when RPCs are placed into a well insert (gray bars), however they are significantly increased in media when RPCs are put into direct contact with PBMCs (white bars, ^*^P < 0.05) when compared to PBMCs in the absence of RPCs (black bars). In contrast, there was no significant difference in IFN-γ release amongst the three groups. Data expressed as mean ± SEM of 2 replicates from 4 kidneys. ^*^p ≤ 0.05 from PBMCs in the absence of RPCs.

To elucidate the potential immunomodulatory properties of RPCs, we performed a mixed leukocyte reaction to examine both direct and paracrine effects of these cells on lymphocytes. As shown in Figure 5, we found a significant effect of RPCs on IL6 release (*P* = 0.05), wherein PHA-stimulated PBMCs released 16.25 ± 0.25 pg/mL in the absence of RPCs, 18.38 ± 0.38 pg/mL when introduced to indirect RPCs, and 19.01 ± 1.08 pg/mL when in direct contact with RPCs. A similar effect was seen with IL8 (*P* = 0.025) with values of 22.88 ± 2.35, 75.19 ± 17.33, and 102.4 ± 42.55 pg/mL being released with PBMCs alone, with indirect RPC contact, and with direct RPC contact, respectively. In contrast to this, we observed a slight decrease in IFN-γ release when RPCs were introduced to the culture; however this difference was not statistically significant.

## Discussion

With over 100,000 patients awaiting a kidney transplant, and another 3,000 added each month, there exists a great need to address more efficient methods of treating renal dysfunction (1). Tissue engineering and regenerative medicine technologies hold immense promise for addressing both AKI and chronic kidney disease (CKD). Recent studies have shown the feasibility of isolating RPCs from adult humans (29). Moreover, their documented (and somewhat surprising) therapeutic efficacy in animal models has generated much excitement for the use of RPCs in renal diseases. As swine models have several advantages in studying renal function, we aimed to determine the regenerative properties of a population of renal progenitors. The primary finding of the study presented herein is that cells isolated from the papillae of porcine kidneys represent a heterogeneous population with RPCs that have limited self-renewing and immunomodulatory capacities.

The limited proliferative ability of porcine RPCs does not preclude their potential use as therapies, as other cell populations or strategies could prove to increase RPC yield. The RPCs isolated herein were from the renal papillae, however other locations may produce a more robust cell line and growth. Bussolati et al. have described a population of RPCs from the cortex of human kidneys that also have a limited ability to proliferate (30). The cells in that study were not able to be passaged more than 7–9 times and exhibited a heterogeneity that we also encountered in the current study (Figure 2). However, populations of RPCs have also been isolated specifically from, for example, proximal (31) and distal (32) tubules, and Bowman's capsule (20) of adult human kidneys (23). Altogether, the limited yield of RPCs from porcine renal papillae may make de- and re-cellularization efforts in porcine kidneys difficult, although worthwhile (33). However, RPCs examined herein were cultured on tissue culture plastic, and there remains the possibility of using substrates such as renal extracellular matrix to promote growth (34).

Despite the diversity in the localizations of different progenitor cells, the majority of these cells are reported to have surface marker expression of CD133 and CD24 (35). Resident CD133+CD24+ cells may play a key role in tubular repair following AKI due to their ability to differentiate into podocyte and tubular lineages (32). Additionally, the number of CD133+ cells is elevated in patients with renal pathologies (36). Isolation of CD24+CD133+ cells from normal human kidneys have been shown to differentiate into tubular, osteogenic, neuronal, and adipocyte cells *in vitro*, and also have the ability to regenerate tubular structures and attenuate kidney damage when injected into immunodeficient mice in acute renal failure (20). As such, these cells represent a viable treatment strategy for conditions of renal dysfunction (37).

Cell surface markers have been at the center of the conversation regarding the different locations of RPCs. While markers such as NFATc1 have been used to illustrate a progenitor cell population from proximal tubules (17), CD24 is one of the consensus markers denoting RPCs (38). RPCs with dual expression of CD24 and CD133 have been shown to possess high regenerative capacity for the treatment of AKI (32, 37), and have the ability to regenerate into podocytes (39). Specifically, CD133+ RPCs cells from renal papilla have been shown to integrate into developing tubules (40). Moreover, CD133+ RPCs promote angiogenesis and erythropoietin production while reducing fibrosis (41). The RPCs in this study expressed CD24 in a passage-dependent manner, indicating senescence might reduce the therapeutic efficacy upon passaging (Figure 3). While flow cytometry did not demonstrate appreciable CD133 expression, the signal with the same antibody was strongly positive in immunocytochemistry, which we attribute to lack of antibody applicability for flow cytometry. This lack of porcine-specific reagents for specific analytical technology is currently a significant obstacle for expanded research into RPCs and/or renal diseases in swine. Moreover, by passage 6 the staining pattern in these cells include areas of a more striated pattern, raising the possibility of non-specific staining with this antibody and highlighting the heterogeneous nature of these cells.

As mentioned in the introduction, the location of the salient RPC population may depend on the underlying cause/etiology of renal dysfunction. For example, it has been shown after ischemia reperfusion injury that tubular regeneration occurs due to cells that are intrinsic to the tubular epithelium (42). Despite this, there remains significant controversy over the phenotype of these cells. One theory suggests that there are resident stem/progenitor cells (also termed “scattered tubular cells”) within the tubular epithelium, while another contends that each tubular epithelial cell has the ability to dedifferentiate and subsequently divide to repair the epithelium (21, 22). There is also evidence showing that tubular RPCs may contribute to tubular repair in the medulla, with the supposition that different regions of the kidney have their own progenitor cell pools (24). While cell sorting methods have been used for isolation of RPCs from these various locations, a recent report displayed a simple primary culture technique from the renal papillae of mice that resulted in a largely homogenous population of cells positive for stem cell markers such as CD90 and CD73 (25). Our efforts in the current study to recapitulate this process using kidneys from large animals resulted in a much more heterogeneous population with mixed positivity for stem cell markers.

The use of swine in kidney disease/transplantation research confers several advantages due to similarities with human kidneys (4). Pig kidneys have a multi-lobed structure similar to humans, with comparable renal size and vascularization. Swine also have similar body sizes which allow for the serial collection of blood samples or kidney biopsies. Pigs have been used in order to optimize the isolation of different RPC populations (43), and have also been used to examine the effectiveness of stem cells in kidney transplant survival (10). Moreover, knockout animals have produced successful xenotransplantation preclinical studies, raising the distinct possibility of renal transplantation from pigs to humans (5). With embryonic human and porcine renal precursors displaying similar ability for nephrogenesis (44), more research needs to be done on the role and efficacy of porcine RPCs.

The immunomodulatory properties of RPCs may eventually dictate their efficacy (or lack thereof) in AKI or other renal diseases. Although comparatively understudied compared to other stem cell types, a series of studies by Huang et al. found that kidney-derived stromal cells from mice have significant immunomodulatory activity (45, 46). The RPCs in that study stimulated bone marrow-derived dendritic cells that exhibited reduced stimulatory effects on CD4+ T cells, which was mediated through cytokine (i.e., IL6, IL1β, and IL12) and vascular endothelial growth factor (VEGF) excretion. Dekel et al. also showed in mice that RPCs lack major histocompatibility complex proteins which may aid in their ability to modulate CD8+ T cells (47). Unfortunately, there remains a paucity of studies that examine RPCs' effects on lymphocytes, especially among larger animal models. While bone marrow MSCs have been shown ameliorate aging in the kidney (27), another study showed a lack of efficacy for porcine bone marrow mesenchymal stem cells (MSC) in reducing AKI (48). This was driven, in part, by their low immunomodulatory activity, with porcine MSCs actually causing increased IL6 production. We found similar results in this study, wherein proinflammatory cytokines IL6 and IL8 were increased when placed into direct contact with porcine lymphocytes (Figure 5). Although this effect was relatively modest, the immunomodulatory activity of RPC populations warrants further investigation, as immunosuppressive activities would advocate for clinical use, while immunogenic activities would prohibit them.

Another consideration is that the isolation of RPCs in this study was from relatively healthy swine (Table 1). The use of normal, healthy, human donor kidneys for the isolation of RPCs might initially seem unnecessary given the growing number of patients on the kidney transplant list. However, RPCs may be isolated from diseased kidneys as well. For example, Gheisari et al. have shown that RPCs can be isolated and expanded from a mouse model of CKD (49), and that these cells are efficacious in a model of cisplatin-induced AKI. Moreover, a recent study also found that human primary cell cultures from both normal and CKD kidneys produced RPCs that had similar proliferative abilities, cell surface markers and phenotype (50). This brings forth the possibility that RPCs isolated from diseased kidneys from patients receiving transplants could be expanded and re-introduced in an autologous fashion to prolong the viability of the transplanted organ. The specific boundary conditions for this type of situation should be examined with preclinical animal models such as swine.

This study has several limitations worth mentioning. As mentioned earlier, this study only examines RPCs from one location within the kidney, and does not examine progenitor cells from, for example, the cortex. While the papillae represents one of the first (and thus extensively) characterized RPC population (19), it is now well recognized that many other such populations exist (24). Additionally, the degree of heterogeneity from subsequent passaging in, for example, differentiation capabilities was not quantified (i.e., Western blotting or RT-PCR). As such, we cannot rule out the possibility that this mixed cell population contains different progenitors with single lineage differentiation capacities. Also, experiments were limited by the relatively small amount of pig reagents and antibodies when compared to humans and rodents. Second, the phenotypic heterogeneity led to a low amount of harvestable cells (and thus biological replicates) for passage 6 which prevented analyses in the mixed lymphocyte reaction. Most importantly, these RPCs were not injected into any renal damage or disease model for examination of their therapeutic efficacy *in vivo*.

In conclusion, the current study shows that RPCs can be isolated from the renal papilla of porcine kidneys. Due to some of the limitations above, it is not clear whether this represents a single, multipotent progenitor population, or several progenitor cell types with differentiation potential. Some heterogeneity is apparent in cell surface marker expression, and differentiation capabilities, however some of these properties are consistent with RPCs from other species. Regardless, the proliferative ability, and “stemness” properties of these cells are passage dependent, and might limit their expansion *ex vivo*. While swine will remain an important preclinical model for renal damage and transplantation, other populations of RPCs within the kidney should be investigated further.

## Disclosure

The opinions or assertions contained herein are the private views of the author and are not to be construed as official or as reflecting the views of the Department of the Army or the Department of Defense.

## Author contributions

All authors participated in the interpretation of the studies and analysis of the data and also reviewed and approved the final version of the manuscript. MM, BG, and RM conducted the experiments; DB and MD were involved in the design of the studies. DB wrote the first draft of the manuscript.

### Conflict of interest statement

The authors declare that the research was conducted in the absence of any commercial or financial relationships that could be construed as a potential conflict of interest.

## References

[1] National Kidney Foundation Organ Donation and Transplantation Statistics (2017). Available online at: https://www.kidney.org/news/newsroom/factsheets/Organ-Donation-and-Transplantation-Stats (Accessed September 30, 2017).

[2] Mundt HM Yard BA Kramer BK Benck U Schnulle P. Optimized donor management and organ preservation before kidney transplantation. Transpl. Int. (2016) 29:974–84. 10.1111/tri.12712 26563531

[3] Tillou X Howden BO Kanellis J Nikolic-Paterson DJ Ma FY. Methods in renal research: kidney transplantation in the rat. Nephrology (Carlton) (2016) 21:451–6. 10.1111/nep.12697 26648592

[4] Giraud S Favreau F Chatauret N Thuillier R Maiga S Hauet T. Contribution of large pig for renal ischemia-reperfusion and transplantation studies: the preclinical model. J Biomed Biotechnol. (2011) 2011:532127. 10.1155/2011/532127 21403881 PMC3051176

[5] Ekser B Rigotti P Gridelli B Cooper DK. Xenotransplantation of solid organs in the pig-to-primate model. Trans Immunol. (2009) 21:87–92. 10.1016/j.trim.2008.10.005 18955143

[6] Waltz E. When pig organs will fly. Nat Biotechnol. (2017) 35:1133–8. 10.1038/nbt.4027 29220027

[7] Chertow GM Burdick E Honour M Bonventre JV Bates DW. Acute kidney injury, mortality, length of stay, and costs in hospitalized patients. J Am Soc Nephrol. (2005) 16:3365–70. 10.1681/ASN.2004090740 16177006

[8] Agarwal A Dong Z Harris R Murray P Parikh SM Rosner MH. Cellular and molecular mechanisms of AKI. J Am Soc Nephrol. (2016) 27:1288–99. 10.1681/ASN.2015070740 26860342 PMC4849836

[9] Burmeister DM Gomez BI Dubick MA. Molecular mechanisms of trauma-induced acute kidney injury: inflammatory and metabolic insights from animal models. Biochim Biophys Acta (2017) 1863(10 Pt B):2661–71. 10.1016/j.bbadis.2017.04.011 28431991

[10] Baulier E Favreau F Le Corf A Jayle C Schneider F Goujon JM. Amniotic fluid-derived mesenchymal stem cells prevent fibrosis and preserve renal function in a preclinical porcine model of kidney transplantation. Stem Cells Transl Med. (2014) 3:809–20. 10.5966/sctm.2013-0186 24797827 PMC4073821

[11] Liu X Cai J Jiao X Yu X Ding X. Therapeutic potential of mesenchymal stem cells in acute kidney injury is affected by administration timing. Acta Biochimica et Biophysica Sinica (2017) 49:338–48. 10.1093/abbs/gmx016 28338909

[12] Peired AJ Sisti A Romagnani P Mesenchymal stem cell-based therapy for kidney disease: a review of clinical *Evid Stem Cells Int*. (2016) 2016:4798639 10.1155/2016/4798639 PMC5046016 27721835

[13] Lee PT Lin HH Jiang ST Lu PJ Chou KJ Fang HC. Mouse kidney progenitor cells accelerate renal regeneration and prolong survival after ischemic injury. Stem Cells (2010) 28:573–84. 10.1002/stem.310 20099318

[14] Lin F Moran A Igarashi P Intrarenal cells, not bone marrow-derived cells, are the major source for regeneration in postischemic kidney. J Clin Invest. (2005) 115:1756–64. 10.1172/JCI23015 16007252 PMC1159127

[15] Bruno S Bussolati B Grange C Collino F di Cantogno LV Herrera MB. Isolation and characterization of resident mesenchymal stem cells in human glomeruli. Stem Cells Dev. (2009) 18:867–80. 10.1089/scd.2008.0320 19579288

[16] Fatima H Moeller MJ Smeets B Yang HC D'Agati VD Alpers CE. Parietal epithelial cell activation marker in early recurrence of FSGS in the transplant. Clin J Am Soc Nephrol. (2012) 7:1852–8. 10.2215/CJN.10571011 22917699 PMC3488951

[17] Langworthy M Zhou B de Caestecker M Moeckel G Baldwin HS. NFATc1 identifies a population of proximal tubule cell progenitors. J Am Soc Nephrol. (2009) 20:311–21. 10.1681/ASN.2008010094 19118153 PMC2637056

[18] Maeshima A Yamashita S Nojima Y. Identification of renal progenitor-like tubular cells that participate in the regeneration processes of the kidney. J Am Soc Nephrol. (2003) 14:3138–46. 10.1097/01.ASN.0000098685.43700.28 14638912

[19] Oliver JA Maarouf O Cheema FH Martens TP Al-Awqati Q. The renal papilla is a niche for adult kidney stem cells. J Clin Invest. (2004) 114:795–804. 10.1172/JCI20921 15372103 PMC516259

[20] Sagrinati C Netti GS Mazzinghi B Lazzeri E Liotta F Frosali F. Isolation and characterization of multipotent progenitor cells from the Bowman's capsule of adult human kidneys. J Am Soc Nephrol. (2006) 17:2443–56. 10.1681/ASN.2006010089 16885410

[21] Kramann R Kusaba T Humphreys BD. Who regenerates the kidney tubule? Nephrol Dialysis Trans. (2015) 30:903–10. 10.1093/ndt/gfu281 25155054 PMC4438740

[22] Lombardi D Becherucci F Romagnani P. How much can the tubule regenerate and who does it? An open question. Nephrol Dialysis Trans. (2016) 31:1243–50. 10.1093/ndt/gfv262 26175143 PMC4967725

[23] Bruno S Chiabotto G Camussi G. Concise review: different mesenchymal stromal/stem cell populations reside in the adult kidney. Stem Cells Transl Med. (2014) 3:1451–5. 10.5966/sctm.2014-0142 25355731 PMC4250217

[24] Oliver JA Sampogna RV Jalal S Zhang QY Dahan A Wang W. A subpopulation of label-retaining cells of the kidney papilla regenerates injured kidney medullary tubules. Stem Cell Rep. (2016) 6:757–71. 10.1016/j.stemcr.2016.03.008 27117784 PMC4939828

[25] Yang G Jia Y Li C Cheng Q Yue W Pei X. Hyperglycemic stress impairs the stemness capacity of kidney stem cells in rats. PLoS ONE (2015) 10:e0139607. 10.1371/journal.pone.0139607 26431335 PMC4592017

[26] Bagul A Frost JH Drage M. Stem cells and their role in renal ischaemia reperfusion injury. Am J Nephrol. (2013) 37:16–29. 10.1159/000345731 23295823

[27] Yang HC Rossini M Ma LJ Zuo Y Ma J Fogo AB. Cells derived from young bone marrow alleviate renal aging. J Am Soc Nephrol. (2011) 22:2028–36. 10.1681/ASN.2010090982 21965376 PMC3231782

[28] Zuk PA Zhu M Mizuno H Huang J Futrell JW Katz AJ. Multilineage cells from human adipose tissue: implications for cell-based therapies. Tissue Eng. (2001) 7:211–28. 10.1089/107632701300062859 11304456

[29] Bussolati B Camussi G. Therapeutic use of human renal progenitor cells for kidney regeneration. Nat Rev Nephrol. (2015) 11:695–706. 10.1038/nrneph.2015.126 26241019

[30] Bussolati B Bruno S Grange C Buttiglieri S Deregibus MC Cantino D. Isolation of renal progenitor cells from adult human kidney. Am J Pathol. (2005) 166:545–55. 10.1016/S0002-944062276-6 15681837 PMC1602314

[31] Lindgren D Bostrom AK Nilsson K Hansson J Sjolund J Moller C. Isolation and characterization of progenitor-like cells from human renal proximal tubules. Am J Pathol. (2011) 178:828–37. 10.1016/j.ajpath.2010.10.026 21281815 PMC3070548

[32] Angelotti ML Ronconi E Ballerini L Peired A Mazzinghi B Sagrinati C. Characterization of renal progenitors committed toward tubular lineage and their regenerative potential in renal tubular injury. Stem Cells (2012) 30:1714–25. 10.1002/stem.1130 22628275

[33] Song JJ Guyette JP Gilpin SE Gonzalez G Vacanti JP Ott HC. Regeneration and experimental orthotopic transplantation of a bioengineered kidney. Nature Med. (2013) 19:646–51. 10.1038/nm.3154 23584091 PMC3650107

[34] O'Neill JD Freytes DO Anandappa AJ Oliver JA Vunjak-Novakovic GV. The regulation of growth and metabolism of kidney stem cells with regional specificity using extracellular matrix derived from kidney. Biomaterials (2013) 34:9830–41. 10.1016/j.biomaterials.2013.09.022 24074840 PMC3835733

[35] Aggarwal S Moggio A Bussolati B. Concise review: stem/progenitor cells for renal tissue repair: current knowledge and perspectives. Stem Cells Transl Med. (2013) 2:1011–9. 10.5966/sctm.2013-0097 24167320 PMC3841081

[36] Loverre A Capobianco C Ditonno P Battaglia M Grandaliano G Schena FP. Increase of proliferating renal progenitor cells in acute tubular necrosis underlying delayed graft function. Transplantation (2008) 85:1112–9. 10.1097/TP.0b013e31816a8891 18431230

[37] Sallustio F Serino G Schena FP. Potential reparative role of resident adult renal stem/progenitor cells in acute kidney injury. Biores Open Access (2015) 4:326–33. 10.1089/biores.2015.0011 26309808 PMC4509615

[38] Challen GA Martinez G Davis MJ Taylor DF Crowe M Teasdale RD. Identifying the molecular phenotype of renal progenitor cells. J Am Soc Nephrol. (2004) 15:2344–57. 10.1097/01.ASN.0000136779.17837.8F 15339983

[39] Ronconi E Sagrinati C Angelotti ML Lazzeri E Mazzinghi B Ballerini L. Regeneration of glomerular podocytes by human renal progenitors. J Am Soc Nephrol. (2009) 20:322–32. 10.1681/ASN.2008070709 19092120 PMC2637058

[40] Ward HH Romero E Welford A Pickett G Bacallao R Gattone VH 2nd. Adult human CD133/1(+) kidney cells isolated from papilla integrate into developing kidney tubules. Biochim Biophys Acta (2011) 1812:1344–57. 10.1016/j.bbadis.2011.01.010 21255643 PMC3166446

[41] Aggarwal S Grange C Iampietro C Camussi G Bussolati B. Human CD133+ renal progenitor cells induce erythropoietin production and limit fibrosis after acute tubular injury. Sci Rep. (2016) 6:37270. 10.1038/srep37270 27853265 PMC5112528

[42] Humphreys BD Valerius MT Kobayashi A Mugford JW Soeung S Duffield JS. Intrinsic epithelial cells repair the kidney after injury. Cell Stem Cell (2008) 2:284–91. 10.1016/j.stem.2008.01.014 18371453

[43] Inowa T Hishikawa K Takeuchi T Kitamura T Fujita T. Isolation and potential existence of side population cells in adult human kidney. Int J Urol. (2008) 15:272–4. 10.1111/j.1442-2042.2007.01984.x 18304230

[44] Dekel B Burakova T Arditti FD Reich-Zeliger S Milstein O Aviel-Ronen S. Human and porcine early kidney precursors as a new source for transplantation. Nat Med. (2003) 9:53–60. 10.1038/nm812 12496960

[45] Huang Y Chen P Zhang CB Ko GJ Ruiz M Fiorina P. Kidney-derived mesenchymal stromal cells modulate dendritic cell function to suppress alloimmune responses and delay allograft rejection. Transplantation (2010) 90:1307–11. 10.1097/TP.0b013e3181fdd9eb 21048532

[46] Huang Y Johnston P Zhang B Zakari A Chowdhry T Smith RR. Kidney-derived stromal cells modulate dendritic and T cell responses. J Am Soc Nephrol. (2009) 20:831–41. 10.1681/ASN.2008030310 19297559 PMC2663835

[47] Dekel B Zangi L Shezen E Reich-Zeliger S Eventov-Friedman S Katchman H. Isolation and characterization of nontubular sca-1+lin- multipotent stem/progenitor cells from adult mouse kidney. J Am Soc Nephrol. (2006) 17:3300–14. 10.1681/ASN.2005020195 17093069

[48] Brunswig-Spickenheier B Boche J Westenfelder C Peimann F Gruber AD Jaquet K. Limited immune-modulating activity of porcine mesenchymal stromal cells abolishes their protective efficacy in acute kidney injury. Stem Cells Dev. (2010) 19:719–29. 10.1089/scd.2009.0494 20143956

[49] Gheisari Y Nassiri SM Arefian E Ahmadbeigi N Azadmanesh K Jamali M. Severely damaged kidneys possess multipotent renoprotective stem cells. Cytotherapy (2010) 12:303–12. 10.3109/14653241003709645 20370347

[50] George SK Abolbashari M Jackson JD Aboushwareb T Atala A Yoo JJ. Potential use of autologous renal cells from diseased kidneys for the treatment of renal failure. PLoS ONE (2016) 11:e0164997. 10.1371/journal.pone.0164997 27776163 PMC5077100

